# The effects of breastfeeding duration on children’s behavior problems at around 3 years of age

**DOI:** 10.3389/fnut.2025.1651419

**Published:** 2025-09-16

**Authors:** Lu Gao, Jianhui Yang, Esben Strodl, Chuanan Wu, Xiaona Yin, Guomin Wen, Dengli Sun, Danxia Xian, Weiqing Chen

**Affiliations:** ^1^Hangzhou Lin’an District Centre for Disease Control and Prevention (Hangzhou Lin’an District Health Supervision Institute), Hangzhou, China; ^2^Ningbo Municipal Centre for Disease Control and Prevention, Ningbo, China; ^3^School of Psychology and Counselling, Queensland University of Technology, Brisbane, QLD, Australia; ^4^Women’s and Children’s Hospital of Longhua District of Shenzhen, Shenzhen, China; ^5^Department of Epidemiology, School of Public Health, Sun Yat-sen University, Shenzhou, China; ^6^Department of Information Management, Xinhua College, Sun Yat-sen University, Guangzhou, China

**Keywords:** breastfeeding patterns, behavioral problems, neurodevelopment, children, China

## Abstract

**Background:**

Although breastfeeding has been demonstrated to benefit children’s health in the initial stages, the lasting effects on behavioral development throughout childhood remain unclear. This study explored the associations between exclusive and overall breastfeeding duration and behavioral problems in Chinese children, aiming to establish evidence-based recommendations for their prevention and management.

**Methods:**

A cross-sectional study involving 17,867 3-year-old children in Longhua District, Shenzhen, China, used questionnaires to collect data on socio-economic status, breastfeeding patterns, and behavioral problems. Breastfeeding durations were analyzed as continuous and categorical variables. Logistic regression, linear regression, and spline plots were used to assess the relationships.

**Results:**

Among 17,867 children, 14.3% had behavioral problems. Exclusive breastfeeding for the first 6 months was significantly associated with lower psychosomatic problem scores. An L-shaped relationship was observed between the duration of exclusive breastfeeding and behavioral problems. Breastfeeding for ≥13 months, compared with ≤6 months, was associated with a lower risk of behavioral problems, particularly impulsive-hyperactivity, and lower scores for learning and psychosomatic issues. Additionally, a linear relationship was observed between total breastfeeding duration and behavioral problems.

**Conclusion:**

Adequate exclusive breastfeeding and extended breastfeeding could reduce behavioral problems in Chinese children, but the causal directionality of observed associations remains undetermined due to cross-sectional data. Given the alignment with current breastfeeding guidelines, interventions to extend breastfeeding duration may help mitigate childhood behavioral problems. Further cohort studies are needed to confirm causality and understand long-term impacts.

## Introduction

Children with behavioral problems are prone to having long-term adverse effects in adulthood (1), making them a persistent and significant topic in pediatrics. Behavioral problems encompass a spectrum of conduct that exceeds the normative bounds for age in terms of duration and intensity (2). Child behavior problems are most often examined under two broad-spectrum dimensions: internalizing problems and externalizing problems. Internalizing problem behaviors encompass withdrawal, anxiety, depression, fear, and compulsions, whereas externalizing problem behaviors include hyperactivity, disobedience, disciplinary infractions, and aggression (3). Globally, it is estimated that between 10 and 20% of children and adolescents experience a range of psychological and behavioral problems (4, 5). Similarly, behavioral problems are common among Chinese children, with estimates ranging from 6 to 26% having at least one behavioral issue (6). Furthermore, the United Nations International Children’s Emergency Fund (UNICEF) has estimated that at least 30 million children and adolescents under the age of 17 in China face behavioral problems (7). Childhood-onset behavioral problems, such as anxiety, depression, attention-deficit disorders, hyperactivity disorders, and aggression, not only impede growth, development, and socialization but may also precipitate learning difficulties, substance abuse, domestic violence, delinquency, antisocial conduct, suicide, and psychopathology during adolescence and adulthood (1, 2, 8–10). These issues impose a substantial burden on families and society, exerting profound negative impacts on individuals, families, and societal structures. The enduring effects of these problems throughout life and their influence on the surrounding milieu often render the mental health needs of adolescents overlooked (4, 11). The occurrence of behavioral problems is the culmination of a multifaceted interplay of factors, adhering to a multi-etiological paradigm. Risk factors include genetics, lifestyle, family dynamics, social environment, and other contributing dimensions (12–14). Therefore, early detection of modifiable factors is essential and urgent for the effective intervention and prevention of these problems in adolescence and adulthood.

Breastfeeding offers a multitude of benefits for both mothers and infants, making it a natural and highly recommended choice for nourishment during the early stages of life. The World Health Organization (WHO) and the UNICEF advocate comprehensive support for women to practice optimal infant and young child feeding (IYCF), such as initiating breastfeeding within 1 h of birth, maintaining exclusive breastfeeding for the initial 6 months (without any other foods or liquids), and continuing breastfeeding for at least 2 years or longer (15). Additionally, they recommended the introduction of adequate, safe, and suitable complementary foods after the first 6 months (16). Breastfeeding offers a range of invaluable benefits exclusively tailored to the wellbeing and development of infants, making it the optimal choice for nourishment during the crucial early years. The advantages of breast milk (BM) for children are profound and multifaceted. It provides critical neurodevelopmental advantages through its unique composition, directly supporting brain growth and function. BM contains essential fatty acids (e.g., DHA), cholesterol, and growth factors that promote neuronal connectivity and myelination, fostering optimal cognitive development (17–19). These nutrients, alongside immune-boosting antibodies, reduce infection risk and inflammation, creating a physiological environment conducive to early brain maturation.

The emotional and social benefits of breastfeeding further shape behavioral outcomes. Skin-to-skin contact during feeding stimulates oxytocin release, strengthening maternal–infant attachment and emotional regulation. This secure bond lays the foundation for healthy socioemotional development, reducing the risk of anxiety and aggression while promoting empathy and social competence (20, 21). Moreover, breastfeeding can have long-term cognitive benefits. Studies have shown that breastfed infants score higher on intelligence tests and demonstrate superior cognitive abilities throughout childhood and adolescence (22). This cognitive edge can be attributed to the unique nutrients and growth factors present in BM that support brain development (19, 23).

Although the benefits of breastfeeding for infants have been studied for many years, there is a significant lack of research looking at the duration of exclusive breastfeeding or breastfeeding and its impact on behavioral problems in preschool children. A few longitudinal and cross-sectional studies have demonstrated the negative association between duration of exclusive breastfeeding and developmental problems in younger children (24). However, a cohort study from China did not find a significant relationship between exclusive breastfeeding and behavioral problems in children (25); a similar association was also found in Japan and Canada (26, 27). Moreover, the majority of previous studies have examined the association between a longer duration of breastfeeding and single or several types of behavioral problems of children (22, 24, 25, 28–30). However, some studies have reported no significant association between a longer breastfeeding duration and the presence of behavioral problems (31). Furthermore, these studies predominantly focused on breastfeeding practices within the first 12 months of life, offering scant information on breastfeeding that extends up to 2 years, as recommended by the WHO. Moreover, these studies treated exclusive breastfeeding and overall breastfeeding duration as categorical variables, overlooking their potential as continuous variables in predicting behavioral development in children. Moreover, the majority of these studies were limited to Caucasian populations, resulting in a significant data gap for Asian populations, especially the Chinese. Therefore, there is a need to further examine the association between the duration of exclusive breastfeeding and the overall duration of breastfeeding with a range of behavioral problems of children in China.

Breastfeeding duration is associated with child neurodevelopment, yet Asian (such as Chinese) populations remain understudied due to Western-centric research biases. Furthermore, previous studies often use logistic regression models treating breastfeeding duration as a categorical variable, thereby obscuring nonlinear dose–response relationships. Gaining deeper insight into the relationship between the duration of exclusive breastfeeding or overall breastfeeding and behavioral problems in children could enable earlier and more impactful interventions, ultimately fostering healthier child development. This study leverages a largely representative sample of Chinese mother–child pairs to address these gaps, employing restricted cubic spline regression to analyze breastfeeding duration as a continuous variable, along with gender stratification analysis. To broaden our understanding of how these breastfeeding patterns influence children’s behavioral problems, the present study endeavors to explore the following inquiries: (1) What is the association between either an extended duration of exclusive breastfeeding or overall breastfeeding and the degree of six behavioral problems (conduct problems, learning problems, psychosomatic problems, impulse-hyperactivity, and anxiety) in Chinese children? and (2) Does gender moderate the associations between duration of exclusive breastfeeding or duration of breastfeeding and the six behavioral problems (conduct problems, learning problems, psychosomatic problems, impulse-hyperactivity, anxiety, and hyperactivity) measured? The results of this study will enrich the existing evidence on the links between breastfeeding patterns and children’s behavioral problems, thereby aiding policymakers, healthcare professionals, educators, and social support workers in formulating and executing effective policies and programs.

## Methods

### Study design and participants

The Longhua Child Cohort Study (LCCS), initiated in Longhua District, Shenzhen, China, in 2014, primarily examines the influence of family and school environments on children’s behavior and mental health. A detailed description of the cohort has been provided elsewhere (32, 33). In September 2019, a total of 18,035 children, approximately 3 years of age, were enrolled in the LCCS after being administered by 171 kindergartens in Longhua District. The baseline data from this study were utilized for our research. After excluding 168 (168/18035 = 0.93%) participants due to mothers not providing complete information, a total of 17,867 (17,867/18035 = 99.07%) child–mother pairs were included in the final data analysis. The study received approval from the Ethics Committee of the School of Public Health at Sun Yat-sen University (2015–16), and written informed consent was obtained from all the children’s mothers.

### Data collection

The mothers of enrolled preschoolers were asked to sign the informed consent and to complete a self-administered structured questionnaire. The questionnaire included questions about parental sociodemographic characteristics (i.e., parental educational level, age at the time of the child’s birth, family income, and marital status), children’s general information (i.e., preterm birth, birth weight, birth length, single child or not, duration of exclusive breastfeeding, and duration of breastfeeding), and behavioral problems.

### Measurement of the breastfeeding duration

The WHO defines the duration of exclusive breastfeeding as the period during which an infant receives only BM, without any supplementary liquids or solid foods. Conversely, the overall duration of breastfeeding refers to the total length of time a mother continues to nurse her child, either with her own BM or with complementary feeding, until weaning (34). In a comprehensive survey, mothers were asked to self-report both the duration of exclusive breastfeeding and the overall duration of breastfeeding by responding to two specific questions tailored to these topics. For statistical analysis, it was important to note that exclusive breastfeeding was treated in two ways: first, as a categorical variable, indicating whether the child was exclusively breastfed during the first 6 months of life, and second, as a continuous variable reflecting the duration of exclusive breastfeeding. Similarly, overall breastfeeding duration was analyzed both as a categorical variable (categorized as ≤6 months, 7–12 months, or ≥13 months) and as a continuous variable. This dual treatment allowed for a nuanced examination of the data, as detailed in the subsequent statistical analysis section.

### Measurement of children’s behavioral problems

The 48-item Conners’ Parent Symptom Questionnaire (PSQ) has undergone multiple revisions since its original development, with the 1978 revision being the most extensively utilized version in China (35). Comprising 48 items, it encompasses six subscales measuring conduct problems, learning problems, psychosomatic problems, impulse-hyperactivity, and anxiety. Furthermore, a hyperactivity index, comprising 10 items particularly sensitive to treatment effects, was also included (36). Each item was rated on a four-point Likert scale ranging from 0 (never) to 3 (very often). The severity of behavioral problems in each domain was assessed by calculating the mean score of all relevant items. Children were defined as having a presence of behavioral problems when any dimension > 2 standard deviations (SDs) above the Chinese normative mean for each gender was observed (35).

### Covariates

We collected data from mothers regarding parental and child demographics, and health information through a self-administered questionnaire measuring child gender, age, birth weight (g), birth length (cm), preterm birth, single child or not, parents’ marital status, education level, family income, parity, parental age at the time of the child’s birth, supplementation time (month), and gestational diseases. We selected these covariates based on relevant information collected from previous relevant publications and questionnaires (11, 22, 24–26, 31).

### Statistical analyses

Continuous variables were presented either as the mean ± standard deviation (SD) or as the median with the interquartile range (IQR), whereas categorical variables were reported using frequencies and percentages. Depending on the data distribution, baseline continuous variables were analyzed using the *t*-test, Mann–Whitney U-test, one-way analysis of variance (ANOVA), or Kruskal-Wallis test. For categorical variables, the chi-square test or Fisher’s exact probability method was employed. Multivariate logistic regression models were utilized to estimate the odds ratios (ORs) and 95% confidence intervals (CIs) for the association between breastfeeding patterns (including durations of exclusive and overall breastfeeding) and the risk of behavioral problems. Additionally, linear regressions were applied to investigate the relationship between breastfeeding patterns and behavioral problem scores. Two models were constructed: Model 1 was unadjusted, while Model 2 was adjusted for the following covariates: child gender, birth weight (g), birth length (cm), preterm birth status, whether the child was an only child, parents’ marital status, education level, family income, parity, parental age at the time of the child’s birth, supplementation time (month), and gestational diseases. Furthermore, restricted cubic spline regression analyses were conducted to assess any potential non-linear relationship between breastfeeding patterns and behavioral problems. The thresholds defining <2 months for exclusive breastfeeding and 8 months for the total breastfeeding time were determined by the results from restricted cubic spline regressions, and then, the two thresholds were dichotomized and examined using logistic regression models to evaluate their effects on behavioral outcomes.

Given the gender-based differences in children’s behavioral problems (37), we conducted a subgroup analysis that categorized children by their gender (boys and girls). To assess whether these associations differed across genders, we incorporated an interaction term into our multivariable regression models.

It is important to note that all statistical evaluations were assessed using two-tailed tests with a significance level of 0.05. All analyses were performed using R (version 4.4.1, http://www.r-project.org).

## Results

### Socio-demographic and obstetric characteristics among participants

Within our sample, 2,555 (2,555/17,867) children, aged around 3 years old, had one of six behavioral problems. The comparison of socio-demographic characteristics between preschoolers without and with behavioral problems is presented in Table 1. Children with behavioral problems were more likely to have a lower birth length than children without behavioral problems. Parents of children with behavioral problems were younger, less educated, had lower yearly family income, and were of single marital status. Furthermore, mothers of children with behavioral problems were more likely to have threatened abortion and gestational diseases (gestational hypertension, preeclampsia/eclampsia, and gestational diabetes mellitus). In addition, children with behavioral problems had shorter exclusive breastfeeding time and overall breastfeeding time than those without behavioral problems (see more details in Table 1). The characteristics of the different sexes are shown in Supplementary Table A5.

**Table 1:** Characteristics of the study samples.

Characteristics	Overall (*n* = 17,867)	Children without behavioral problems (*n* = 15,312)	Children with behavioral problems (*n* = 2,555)	*p*
Child gender (%)				0.250
Boy	9,648 (54.0)	8,241 (53.8)	1,407 (55.1)	
Girl	8,219 (46.0)	7,071 (46.2)	1,148 (44.9)	
Child age [mean (SD)]	3.48 (0.27)	3.48 (0.27)	3.47 (0.26)	0.061
Birth weight [mean (SD)]	3401.09 (864.35)	3404.29 (864.10)	3381.90 (865.78)	0.226
Birth length [mean (SD)]	50.95 (5.55)	51.01 (5.50)	50.60 (5.86)	0.001
Maternal age at child birth [mean (SD)]	29.23 (4.20)	29.32 (4.21)	28.64 (4.11)	<0.001
Paternal age at child birth [mean (SD)]	34.80 (4.68)	34.90 (4.68)	34.17 (4.64)	<0.001
Maternal education level				<0.001
Junior high school or lower	4,835 (27.1)	4,077 (26.6)	758 (29.7)	
High school	7,340 (41.1)	6,271 (41.0)	1,069 (41.8)	
College or higher	5,692 (31.9)	4,964 (32.4)	728 (28.5)	
Paternal education level				<0.001
Junior high school or lower	4,529 (25.3)	3,827 (25.0)	702 (27.5)	
High school	6,237 (34.9)	5,313 (34.7)	924 (36.2)	
College or higher	7,101 (39.7)	6,172 (40.3)	929 (36.4)	
Family income (RMB/month)				<0.001
<5,000	4,102 (23.0)	3,463 (22.6)	639 (25.0)	
5,001–10,000	6,404 (35.8)	5,445 (35.6)	959 (37.5)	
10,001–20,000	3,870 (21.7)	3,329 (21.7)	541 (21.2)	
>20,000	3,491 (19.5)	3,075 (20.1)	416 (16.3)	
Marital status				0.007
Married	17,353 (97.1)	14,896 (97.3)	2,457 (96.2)	
Single	514 (2.9)	380 (2.7)	134 (3.8)	
Single child or not				<0.001
No	10,014 (56.0)	8,730 (57.0)	1,284 (50.3)	
Yes	7,853 (44.0)	6,582 (43.0)	1,271 (49.7)	
Threatened abortion				0.012
No	15,420 (86.3)	13,256 (86.6)	2,164 (84.7)	
Yes	2,447 (13.7)	2056 (13.4)	391 (15.3)	
Gestational hypertension				<0.001
No	17,472 (97.8)	14,998 (97.9)	2,474 (96.8)	
Yes	395 (2.2)	314 (2.1)	81 (3.2)	
Preeclampsia/eclampsia				<0.001
No	17,769 (99.5)	15,250 (99.6)	2,519 (98.6)	
Yes	98 (0.5)	62 (0.4)	36 (1.4)	
Gestational diabetes mellitus				0.066
No	16,572 (92.8)	14,225 (92.9)	2,347 (91.9)	
Yes	1,295 (7.2)	1,087 (7.1)	208 (8.1)	
Preterm birth				1.000
No	16,304 (91.3)	13,973 (91.3)	2,331 (91.2)	
Yes	1,563 (8.7)	1,339 (8.7)	224 (8.8)	
Time of complementary food [mean (SD)]	5.97 (2.36)	5.97 (2.31)	6.01 (2.60)	0.381
The duration of exclusive breastfeeding [mean (SD)]	3.30 (3.14)	3.33 (3.14)	3.18 (3.14)	0.027
Exclusive breastfeeding in the first 6 months of life				0.156
No	13,347 (74.7)	11,409 (74.5)	1938 (75.9)	
Yes	4,520 (25.3)	3,903 (25.5)	617 (24.1)	
The duration of breastfeeding [mean (SD)]	8.70 (5.72)	8.76 (5.75)	8.31 (5.57)	<0.001
Overall breastfeeding time				0.001
≤6 months	7,015 (39.3)	5,951 (38.9)	1,064 (41.6)	
7–12 months	7,375 (41.3)	6,318 (41.3)	1,057 (41.4)	
≥13 months	3,477 (19.5)	3,043 (19.9)	434 (17.0)	

### Differences in behavioral problems in children with different breastfeeding patterns

Table 2 presents the comparison of children’s behavioral problems by their status of exclusive breastfeeding in the first 6 months of life. We found that, compared to children who were not exclusively breastfed during this period, those who were had lower levels of psychosomatic problems (3.8% vs. 3.1%, *p* = 0.038) and lower scores in three scales of PSQ (psychosomatic problem scores: 0.09 vs. 0.08, *p* = 0.010; impulsive–hyperactive scores: 0.33 vs. 0.31, *p* = 0.007; and hyperactivity index scores: 0.37 vs. 0.36, *p* = 0.033) (see more details in Table 2). Furthermore, logistic regression analysis revealed significant inverse associations between prolonged overall breastfeeding time and the prevalence of behavioral problems, psychosomatic problems, impulsive–hyperactive, and hyperactivity index (all *p* for trend <0.05). In parallel, linear regression models demonstrated that extended overall breastfeeding time was negatively correlated with the severity scores of psychosomatic problems, impulsive–hyperactive symptoms, and hyperactivity index (all *p* for trend < 0.05) (see more details in Table 3).

**Table 2:** Comparison of children’s behavioral problems by their status of exclusive breastfeeding in the first 6 months of life.

Scales	Overall (*n* = 17,867)	exclusive breastfeeding in the first 6 months of life		*p*
		No (*n* = 13,347)	Yes (*n* = 4,520)	
Behavioral problems (%)				0.156
No	15,312 (85.7)	11,409 (85.5)	3,903 (86.3)	
Yes	2,555 (14.3)	1938 (14.5)	617 (13.7)	
Conduct problem (%)				0.456
No	17,269 (96.7)	12,892 (96.6)	4,377 (96.8)	
Yes	598 (3.3)	455 (3.4)	143 (3.2)	
Learning problem (%)				0.149
No	17,061 (95.5)	12,727 (95.4)	4,334 (95.9)	
Yes	806 (4.5)	620 (4.6)	186 (4.1)	
Psychosomatic problem (%)				0.038
No	17,227 (96.4)	12,846 (96.2)	4,381 (96.9)	
Yes	640 (3.6)	501 (3.8)	139 (3.1)	
Impulsive–hyperactive (%)				0.593
No	17,113 (95.8)	12,777 (95.7)	4,336 (95.9)	
Yes	754 (4.2)	570 (4.3)	184 (4.1)	
Anxiety (%)				0.099
No	17,263 (96.6)	12,878 (96.5)	4,385 (97.0)	
Yes	604 (3.4)	469 (3.5)	135 (3.0)	
Hyperactivity index (%)				0.242
No	17,744 (99.3)	13,249 (99.3)	4,495 (99.4)	
Yes	123 (0.7)	98 (0.7)	25 (0.6)	
Conduct problem scores [mean (SD)]	0.36 (0.31)	0.36 (0.31)	0.35 (0.30)	0.156
Learning problem scores (mean (SD))	0.42 (0.40)	0.42 (0.40)	0.41 (0.39)	0.241
Psychosomatic problem scores [mean (SD)]	0.09 (0.18)	0.09 (0.18)	0.08 (0.17)	0.010
Impulsive–hyperactive scores [mean (SD)]	0.32 (0.40)	0.33 (0.40)	0.31 (0.39)	0.007
Anxiety scores [mean (SD)]	0.29 (0.28)	0.29 (0.28)	0.28 (0.27)	0.082
Hyperactivity index scores [mean (SD)]	0.37 (0.34)	0.37 (0.35)	0.36 (0.34)	0.033

*x*^2^ tests were used for categorical variables, and *t*-tests were used for continuous variables.

The reference group was children who were not exclusively breastfed for the first 6 months.

**Table 3:** Comparison of children’s behavioral problems by their status of overall breastfeeding time.

Scales	Overall (*n* = 17,867)	Overall breastfeeding time			*p*	*p* for trend
		≤6 months	7–12 months	≥13 months		
Behavioral problems (%)					0.001	<0.001
No	15,312 (85.7)	5,951 (84.8)	6,318 (85.7)	3,043 (87.5)		
Yes	2,555 (14.3)	1,064 (15.2)	1,057 (14.3)	434 (12.5)		
Conduct problem (%)					0.251	0.096
No	17,269 (96.7)	6,763 (96.4)	7,133 (96.7)	3,373 (97.0)		
Yes	598 (3.3)	252 (3.6)	242 (3.3)	104 (3.0)		
Learning problem (%)					0.540	0.483
No	17,061 (95.5)	6,696 (95.5)	7,033 (95.4)	3,332 (95.8)		
Yes	806 (4.5)	319 (4.5)	342 (4.6)	145 (4.2)		
Psychosomatic problem (%)					0.027	0.007
No	17,227 (96.4)	6,736 (96.0)	7,117 (96.5)	3,374 (97.0)		
Yes	640 (3.6)	279 (4.0)	258 (3.5)	103 (3.0)		
Impulsive–hyperactive (%)					0.001	<0.001
No	17,113 (95.8)	6,683 (95.3)	7,065 (95.8)	3,365 (96.8)		
Yes	754 (4.2)	332 (4.7)	310 (4.2)	112 (3.2)		
Anxiety (%)					0.065	0.021
No	17,263 (96.6)	6,756 (96.3)	7,128 (96.7)	3,379 (97.2)		
Yes	604 (3.4)	259 (3.7)	247 (3.3)	98 (2.8)		
Hyperactivity index (%)					0.012	0.004
No	17,744 (99.3)	6,955 (99.1)	7,324 (99.3)	3,465 (99.7)		
Yes	123 (0.7)	60 (0.9)	51 (0.7)	12 (0.3)		
Conduct problem scores [mean (SD)]	0.36 (0.31)	0.36 (0.31)	0.36 (0.31)	0.35 (0.30)	0.122	0.076
Learning problem scores [mean (SD)]	0.42 (0.40)	0.42 (0.40)	0.42 (0.40)	0.40 (0.39)	0.044	0.048
Psychosomatic problem scores [mean (SD)]	0.09 (0.18)	0.09 (0.19)	0.08 (0.18)	0.08 (0.17)	0.019	0.005
Impulsive–hyperactive scores [mean (SD)]	0.32 (0.40)	0.33 (0.40)	0.33 (0.40)	0.29 (0.37)	<0.001	<0.001
Anxiety scores [mean (SD)]	0.29 (0.28)	0.29 (0.29)	0.28 (0.28)	0.28 (0.28)	0.215	0.084
Hyperactivity index scores [mean (SD)]	0.37 (0.34)	0.38 (0.35)	0.37 (0.35)	0.35 (0.33)	0.003	0.003

The reference group was children who were not breastfed for the first ≤6 months.

### Associations between exclusive breastfeeding for the first 6 months and children’s behavioral problems

The associations between exclusive breastfeeding in the first 6 months of life and children’s behavioral problems are shown in Table 4. After adjusting for confounders, there were no significant relationships between exclusive breastfeeding and any measure of children’s behavioral problems.

**Table 4:** Associations of exclusive breastfeeding in the first 6 months of life with children’s behavioral problems.

Items	cOR (95%CI)	*p*	aOR (95%CI)	*p*
Behavioral problems	0.931 (0.844, 1.026)	0.149	0.944 (0.856, 1.042)	0.257
Conduct problem	0.926 (0.765, 1.121)	0.428	0.948 (0.782, 1.150)	0.589
Learning problem	0.881 (0.745, 1.042)	0.138	0.872 (0.737, 1.032)	0.111
Psychosomatic problem	0.814 (0.672, 0.985)	0.034	0.851 (0.701, 1.032)	0.102
Impulsive–hyperactive	0.951 (0.803, 1.127)	0.564	1.001 (0.843, 1.189)	0.986
Anxiety	0.845 (0.696, 1.027)	0.090	0.870 (0.714, 1.059)	0.164
Hyperactivity index	0.752 (0.484, 1.168)	0.205	0.788 (0.505, 1.229)	0.292

Adjusted for child gender, birth weight (g), birth length (cm), preterm birth status, whether the child was an only child, parents’ marital status, education level, family income, parity, parental age at the time of the child’s birth, supplementation time (month), and gestational diseases (gestational hypertension, preeclampsia/eclampsia and gestational diabetes mellitus).

Table 5 presents the associations of exclusive breastfeeding in the first 6 months of life and children’s PSQ score. After adjusting for confounders, multivariate logistic regression models showed that psychosomatic problem scores were significantly lower in children who were exclusively breastfed during the first 6 months of life (aβ = −0.006, 95%CI: −0.012, −0.001) compared with those who were not.

**Table 5:** Associations of exclusive breastfeeding in the first 6 months of life with children’s PSQ scores.

Items	cβ (95%CI)	*p*	aβ (95%CI)	*p*
Conduct problem scores	−0.007 (−0.018, 0.003)	0.156	−0.005 (−0.015, 0.006)	0.371
Learning problem scores	−0.008 (−0.021, 0.005)	0.241	−0.009 (−0.022, 0.004)	0.192
Psychosomatic problem scores	−0.008 (−0.014, −0.002)	0.010	−0.006 (−0.012, −0.001)	0.047
Impulsive–hyperactive scores	−0.018 (−0.032, −0.005)	0.007	−0.012 (−0.025, 0.001)	0.072
Anxiety scores	−0.008 (−0.018, 0.001)	0.082	−0.008 (−0.018, 0.001)	0.088
Hyperactivity index scores	−0.013 (−0.024, −0.001)	0.033	−0.008 (−0.02, 0.003)	0.149

Adjusted for child gender, birth weight (g), birth length (cm), preterm birth status, whether the child was an only child, parents’ marital status, education level, family income, parity, parental age at the time of the child’s birth, supplementation time (month), and gestational diseases (gestational hypertension, preeclampsia/eclampsia and gestational diabetes mellitus).

**Figure 1:**
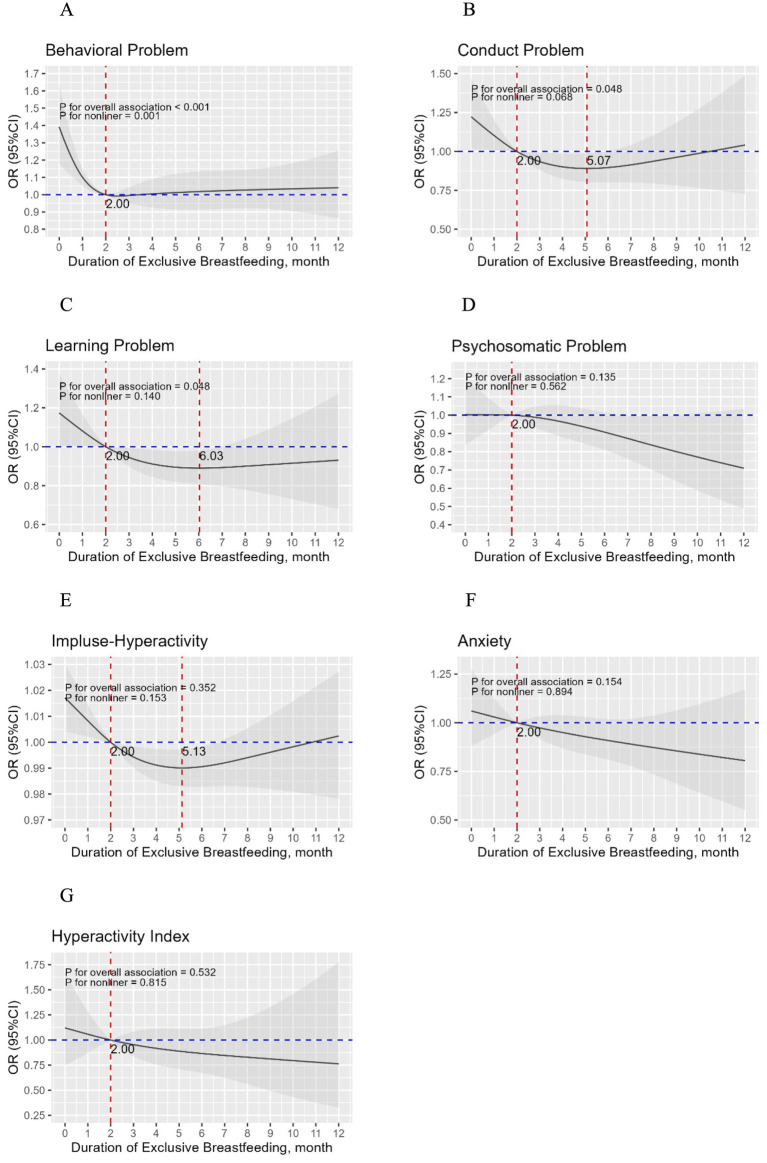
The full-adjusted relationship between duration of exclusive breastfeeding and children’s behavioral problems, sub-scales of behavioral problems. Adjusted for child gender, birth weight (g), birth length (cm), preterm birth status, whether the child was an only child, parents’ marital status, education level, family income, parity, parental age at the time of the child’s birth, supplementation time (month), and gestational diseases (gestational hypertension, preeclampsia/eclampsia and gestational diabetes mellitus).

The non-linear relationship between the duration of exclusive breastfeeding and the risk of children’s behavioral problems exhibited an L-shaped curve (*p* for overall <0.001; *p* for nonlinearity = 0.001), and the likelihood of behavioral problems in the children decreased from 0 months of exclusive breastfeeding to 2 months of exclusive breastfeeding (Figure 1A). In addition, Figures 1B–G show the adjusted correlations between the duration of exclusive breastfeeding and the risk of different subscales of behavioral problems among children. The risks of conduct problems, learning problems, and impulse-hyperactivity in children decreased from 2 months of exclusive breastfeeding to 5.07, 6.03, and 5.13 months of exclusive breastfeeding, respectively (Figures 1B,C,E). Given that the higher probability of behavioral problems among children occurred within <2 months of exclusive breastfeeding, a logistic regression model was constructed with <2 months as the reference group (Table 6). Children who were exclusively breastfed for ≥2 months were less prone to developing behavioral problems compared with those breastfed exclusively for <2 months.

**Table 6:** Logistic regression table of the relationship between breastfeeding time and behavioral problems in children.

Variable	cOR (95%CI)	*p*	aOR (95%CI)	*p*
Exclusive breastfeeding time				
<2 months	1.000 (Reference)		1.000 (Reference)	
≥2 months	0.846 (0.778, 0.921)	<0.001	0.855 (0.785, 0.930)	<0.001
Overall breastfeeding time				
<8 months	1.000 (Reference)		1.000 (Reference)	
≥8 months	0.890 (0.818, 0.968)	0.007	0.912 (0.837, 0.993)	0.0334

Adjusted for child gender, birth weight (g), birth length (cm), preterm birth status, whether the child was an only child, parents’ marital status, education level, family income, parity, parental age at the time of the child’s birth, supplementation time (month), and gestational diseases (gestational hypertension, preeclampsia/eclampsia and gestational diabetes mellitus).

### Associations between the overall breastfeeding time and children’s behavioral problems

We also evaluated the association of the overall breastfeeding time with children’s behavioral problems (Table 7). With the inclusion of confounders into the models, those who were breastfed for 7–12 months had no significant differences in behavioral problems, whereas those who were breastfed for ≥13 months were associated with a lower risk of behavioral problems (aOR = 0.843, 95%CI: 0.746, 0.951), conduct problems (aOR = 1.43, 95%CI: 1.17, 1.75), impulsive–hyperactive (aOR = 0.757, 95%CI: 0.607, 0.945), and hyperactivity index (aOR = 0.451, 95%CI: 0.241, 0.844) compared with counterparts only breastfeeding for ≤6 months.

**Table 7:** Associations of overall breastfeeding time with children’s behavioral problems.

Items	7–12 months (*n* = 6,318)				≥13 months (*n* = 3,043)			
	cOR (95%CI)	*p*	aOR (95%CI)	*p*	cOR (95%CI)	*p*	aOR (95%CI)	*p*
Behavioral problems	0.936 (0.853, 1.026)	0.158	0.938 (0.855, 1.030)	0.194	0.798 (0.708, 0.899)	< 0.001	0.843 (0.746, 0.951)	0.007
Conduct problem	0.911 (0.761, 1.090)	0.306	0.936 (0.780, 1.122)	0.475	0.827 (0.656, 1.044)	0.110	0.917 (0.724, 1.160)	0.469
Learning problem	1.021 (0.873, 1.193)	0.797	1.022 (0.874, 1.196)	0.785	0.913 (0.747, 1.116)	0.376	0.922 (0.753, 1.129)	0.433
Psychosomatic problem	0.875 (0.737, 1.040)	0.130	0.898 (0.754, 1.070)	0.229	0.737 (0.586, 0.927)	0.009	0.794 (0.629, 1.003)	0.053
Impulsive–hyperactive	0.883 (0.754, 1.035)	0.124	0.900 (0.767, 1.057)	0.198	0.670 (0.539, 0.833)	<0.001	0.757 (0.607, 0.945)	0.014
Anxiety	0.904 (0.757, 1.079)	0.264	0.927 (0.774, 1.109)	0.443	0.757 (0.597, 0.958)	0.020	0.822 (0.647, 1.045)	0.139
Hyperactivity index	0.807 (0.555, 1.174)	0.263	0.833 (0.570, 1.217)	0.345	0.401 (0.216, 0.747)	0.004	0.451 (0.241, 0.844)	0.013

Adjusted for child gender, birth weight (g), birth length (cm), preterm birth status, whether the child was an only child, parents’ marital status, education level, family income, parity, parental age at the time of the child’s birth, supplementation time (month), and gestational diseases (gestational hypertension, preeclampsia/eclampsia and gestational diabetes mellitus).

Multivariate logistic regression models showed that only breastfeeding for periods of ≥13 months had significantly lower scores of learning problem (aβ = −0.019, 95%CI: −0.036, −0.003), scores of psychosomatic problem (aβ = −0.008, 95%CI: −0.015, 0), and scores of impulsive–hyperactive (aβ = −0.025, 95%CI: −0.040, −0.009) compared with breastfeeding for ≤6 months (Table 8). No other PSQ scores were significantly associated with duration of breastfeeding.

**Table 8:** Associations of overall breastfeeding time with children’s PSQ scores.

Items	7–12 months (*n* = 6,318)				≥13 months (*n* = 3,043)			
	cβ (95%CI)	*p*	aβ (95%CI)	*p*	cβ (95%CI)	*p*	aβ (95%CI)	*p*
Conduct problem scores	−0.001 (−0.011, 0.009)	0.801	−0.002 (−0.012, 0.008)	0.715	−0.012 (−0.025, 0)	0.050	−0.004 (−0.016, 0.009)	0.559
Learning problem scores	0 (−0.013, 0.013)	0.980	−0.001 (−0.014, 0.012)	0.933	−0.019 (−0.035, −0.003)	0.023	−0.019 (−0.036, −0.003)	0.020
Psychosomatic problem scores	−0.005 (−0.011, 0.001)	0.114	−0.004 (−0.010, 0.002)	0.180	−0.01 (−0.018, −0.003)	0.006	−0.008 (−0.015, 0)	0.038
Impulsive–hyperactive scores	−0.007 (−0.019, 0.006)	0.323	−0.008 (−0.020, 0.005)	0.245	−0.04 (−0.056, −0.024)	<0.001	−0.025 (−0.040, −0.009)	0.003
Anxiety scores	−0.006 (−0.015, 0.003)	0.199	−0.007 (−0.017, 0.002)	0.117	−0.009 (−0.021, 0.002)	0.106	−0.005 (−0.016, 0.007)	0.438
Hyperactivity index scores	−0.003 (−0.014, 0.009)	0.642	−0.004 (−0.015, 0.007)	0.465	−0.023 (−0.037, −0.009)	0.001	−0.012 (−0.025, 0.002)	0.102

Adjusted for child gender, birth weight (g), birth length (cm), preterm birth status, whether the child was an only child, parents’ marital status, education level, family income, parity, parental age at the time of the child’s birth, supplementation time (month), and gestational diseases (gestational hypertension, preeclampsia/eclampsia and gestational diabetes mellitus).

**Figure 2:**
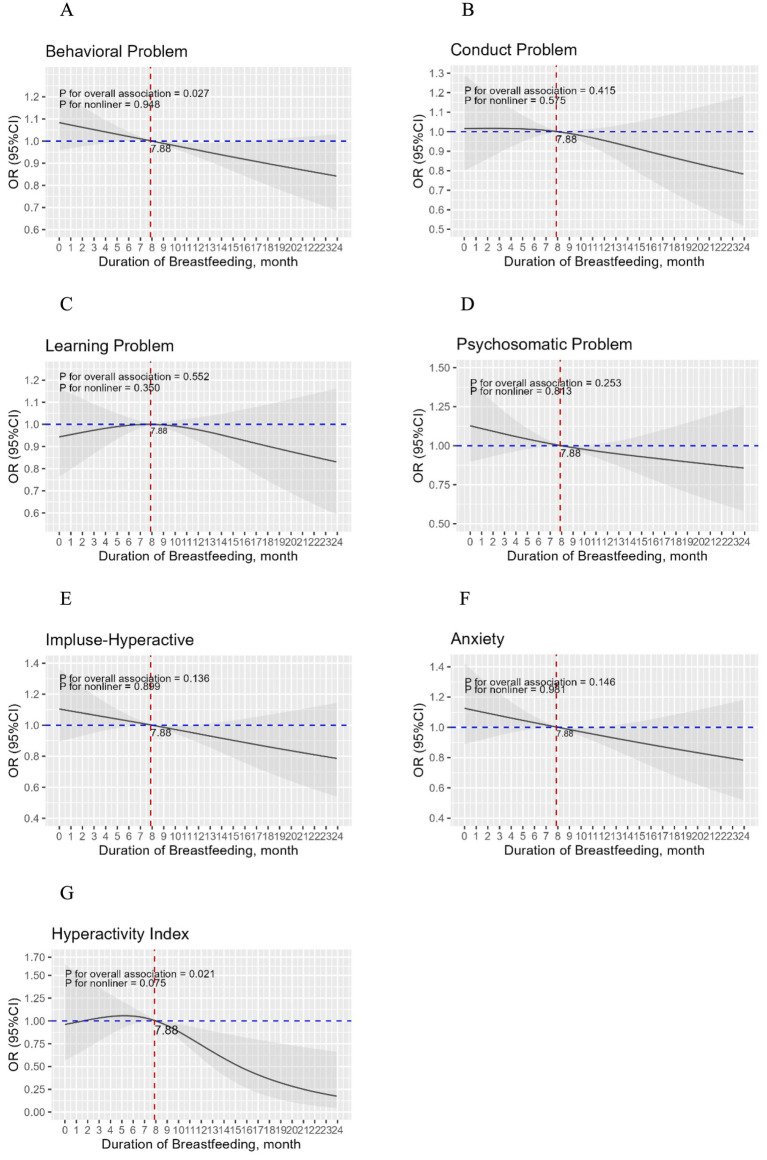
The full-adjusted relationship between duration of breastfeeding and children’s behavioral problems, sub-scales of behavioral problems. Adjusted for child gender, birth weight (g), birth length (cm), preterm birth status, whether the child was an only child, parents’ marital status, education level, family income, parity, parental age at the time of the child’s birth, supplementation time (month), and gestational diseases (gestational hypertension, preeclampsia/eclampsia and gestational diabetes mellitus).

Using restricted cubic splines, linear relationships between the duration of breastfeeding and the risk of children’s behavioral problems were found after adjusting for multiple covariates (Figure 2). Moreover, a threshold effect was observed, with an inflection point at approximately 8 months of breastfeeding. When the duration of breastfeeding months was less than this cutoff, the risk of behavioral problems remained almost unchanged or slightly increased; however, when the duration exceeded the cutoff value, the risk decreased rapidly. Considering that the higher probability of behavioral problems occurred with <8 months of breastfeeding duration, a logistic regression model was constructed with <8 months as the reference group (Table 6). Compared with children with <8 months of breastfeeding duration, those with ≥8 months exhibited a lower likelihood of behavioral problems.

### Subgroup analysis based on the children’s gender

When we conducted a series of subgroup analyses stratified by children’s gender, we found that the significant correlations between exclusive breastfeeding for the first 6 months and overall duration of breastfeeding with behavioral problems persisted only among boys, not girls. Specifically, boys who were exclusively breastfed for the first 6 months exhibited a lower risk of behavioral problems and learning problems compared with those who were not (as shown in Supplementary Table A1). However, no statistically significant differences were observed in their behavioral problem scores or sub-scale scores (Supplementary Table A2). Regarding the overall breastfeeding duration, boys who were breastfed for at least 12 months were less likely to experience behavioral and learning problems (Supplementary Table A3). Furthermore, these boys scored lower on measures of learning and psychosomatic problems, impulsivity–hyperactivity, and anxiety (Supplementary Table A4).

## Discussion

In this study, the final data set included 17,867 Chinese children aged approximately 3 years for analysis, including 9,648 boys and 8,219 girls. Of these, 2,555 children had behavioral problems. Compared with those who were breastfed for <6 months, children who were breastfed for ≥13 months were markedly correlated with better behavioral outcomes. Moreover, after adjusting for all covariates, we found that the relationship between duration of exclusive breastfeeding and behavioral problems was L-shaped, but the association between duration of breastfeeding and behavioral problems was linear. Furthermore, we found that when the duration of exclusive breastfeeding was shorter than 2 months or the total breastfeeding duration was less than 8 months, the risk of behavioral problems increased significantly. Finally, subgroup analyses indicated that these beneficial associations were more pronounced among boys compared with girls. Collectively, these results suggest that optimizing exclusive breastfeeding duration and extending overall breastfeeding duration may exert protective effects against behavioral problems in childhood development.

The evidence regarding the association between exclusive breastfeeding and children’s behavioral problems has been characterized by limited and inconsistent findings. Some studies have reported significantly lower risk of behavioral problems in children with exclusive breastfeeding for the first 6 months (24), whereas others have found no association between exclusive breastfeeding for the first 4 months and children’s behavioral problems (26, 27, 31). However, our study revealed an L-shaped relationship between exclusive breastfeeding and the likelihood of children’s behavioral problems and identified the optimal exclusive breastfeeding duration interval for children as 2–6 months, with approximately 5 months demonstrating the greatest health benefits. This has not been identified in previous studies. Our findings, therefore, may help explain the previous inconsistent findings and support the need for further prospective cohort studies with precise measures of exclusive breastfeeding duration to test the causal direction of this association.

Overall, we found that a longer duration of breastfeeding was associated with better childhood behavioral outcomes, whether measured continuously or categorically. This finding aligns with previous research indicating that long (≥ 6 months) breastfeeding duration was beneficial to behavioral outcomes (29, 38–40). For example, a cross-sectional study conducted in Xiamen with 1,979 children aged between 6 and 11  years has found that increased duration of breastfeeding (≥6 months) was negatively associated with internalizing behavioral problems such as depression and somatic complaints (29). Another UK birth cohort study of children aged between 3 and 14  years has also demonstrated significant associations between breastfeeding duration and reduced parent-reported Strengths and Difficulties Questionnaire (SDQ) scores (40). Our study, however, extends the findings from these previous studies by identifying a new critical duration for the beneficial effects of breastfeeding. An inflection point was observed at approximately 8 months, and when breastfeeding lasts longer than 8 months, the risk of developing behavioral problems was significantly reduced. These findings underscore the critical importance of maintaining optimal breastfeeding duration for better behavioral outcomes.

Several potential mechanisms may elucidate the connections between breastfeeding practices and behavioral problems of children. BM is crucial for early central nervous system development. Key components such as 2′-fucosyllactose (2′-FL) enhance cognitive function in animals and positively impact infants’ cognitive development during the first 6 months (41–43). Sialic acid from sialyllactose crosses the blood–brain barrier, accumulating in brain gangliosides and glycoproteins (44). Human milk oligosaccharides (HMOs) in BM protect against stressors, regulate gut microbiota, and maintain normal neuron numbers (45, 46). Magnetic resonance imaging (MRI) studies show greater white matter development in brain regions linked to behavior and cognition in infants breastfed for at least 3 months (47, 48). BM’s phospholipids, including choline sphingomyelin and phosphatidylcholine, are vital for memory, cognitive and behavioral function, and brain development, significantly increasing sphingomyelin and choline levels in the brain compared with formula milk (49, 50). Additionally, cholesterol, long-chain polyunsaturated fatty acids (LCPUFAs), and docosahexaenoic acid (DHA) in BM benefit preterm infants’ brain development by increasing gray matter volume and enhancing brain activation (51, 52). Moreover, BM’s milk fat globule membrane (MFGM) optimizes cognitive, mental, and behavioral functions, while non-nutritive components such as HMOs, lactoferrin, and microbial species also contribute to early brain development (53–55). Breastfeeding also fosters a unique bond between mother and infant, promoting positive behavioral outcomes in children (25). In essence, BM’s rich nutritional content, bioactive components, and nurturing bond between mother and child are indispensable for infants’ holistic growth and development.

One additional novel observation was the gender difference emerging from our study. That is, we showed negative independent associations between breastfeeding durations and behavioral outcomes in boys but not in girls. This is an important finding given that the analysis of gender as a moderator has been frequently neglected in the literature examining the effect of breastfeeding on developmental outcomes. As such, our findings point to the need to understand the underlying mechanisms resulting in such gender differences. For example, it has been established that behavioral development during fetal and subsequent life is influenced by the consumption of human milk during early life (56–58). However, boys may be more sensitive to nutrients in breast milk early in life due to the “male disadvantage.” (59) In addition, there appear to be gender differences in the composition of maternal milk (59, 60), so the benefits of breastfeeding may be more pronounced for the behavioral development in boys. However, the mechanisms of gender differences in the effects of BM on behavioral problems are currently unclear and need to be investigated to better understand this phenomenon.

A significant advantage of this study lies in its substantial sample size, which greatly enhances the statistical power and reliability of the results. Furthermore, the analysis of exclusive breastfeeding time and breastfeeding time was conducted with both categorical and continuous measures, providing a nuanced understanding of their impact. However, in addition to recognizing the strengths of this study, it is also important to acknowledge its limitations. First, these results are based on cross-sectional data and do not allow us to determine the causal direction of the observed associations. Second, the limitations of existing databases make it difficult to fully include all biological and environmental factors associated with breastfeeding duration, which may partially affect the comprehensiveness and accuracy of the findings. At the same time, the determination of behavioral problems was based on standardized questionnaires completed by parents, rather than including clinical examinations, thus raising the possibility of reporting bias. Additionally, we did not collect data on maternal mental health, like postpartum anxiety, which might affect both feeding practices and children’s behavioral outcomes. Furthermore, our study excluded participants with incomplete information, which may introduce selection bias. However, given the small number of subjects in this group, this exclusion was unlikely to substantially affect the results. Finally, all participants were recruited from the Longhua District of Shenzhen, which may limit the generalizability of our findings. Therefore, further research is required to replicate our findings in other populations using prospective cohort studies that incorporate objective measures of the child’s behavioral problems assessed in this study, together with a wider range of potential covariates.

## Conclusion

In this cross-sectional study involving 17,867 participants, we found that associations between both the duration of exclusive breastfeeding and the duration of overall breastfeeding with children’s behavioral problems after controlling for potential confounders. The duration of exclusive breastfeeding had an L-shaped relationship with the likelihood of behavioral problems, and the duration of breastfeeding had a linear relationship with the likelihood of behavioral problems. As such, the likelihood of behavioral problems in children may be reduced by increasing the duration of exclusive breastfeeding within the optimal exclusive breastfeeding range or duration of breastfeeding identified in this study. Further longitudinal designs with birth cohorts across different populations and areas (urban and rural) are warranted to precisely elucidate these causal relationships and explore the underlying mechanisms.

## Data Availability

The original contributions presented in the study are included in the article/Supplementary material, and further inquiries can be directed to the corresponding authors.
