# Supplementary material for: The effects of breastfeeding duration on children’s behavior problems at around 3 years of age
Source: Front Nutr. 2025 Sep 16;12:1651419. doi: 10.3389/fnut.2025.1651419 (PMC12479251; doi:10.3389/fnut.2025.1651419)
Supplement: Supplementary file 1 [file Table_1.DOCX]

**TableA 1 Risks of children’s behavioral problems based on feeding pattern (exclusive breastfeeding vs. non-** **exclusive breastfeeding) for first 6 months stratified by children’s sex^*†^.**

| **Items** | **Boys (9648)** | | | **Girls (8219)** | | |
| --- | --- | --- | --- | --- | --- | --- |
|  | **Events** | **aOR (95%CI)** | ***P*** | **Events** | **aOR (95%CI)** | ***P*** |
| **Behavioral problems^#^** | 312 (22.2%) | 0.863 (0.752, 0.989) | 0.035 | 305 (26.6%) | 1.045 (0.905, 1.206) | 0.548 |
| **Conduct problem** | 56 (20.4%) | 0.793 (0.587, 1.070) | 0.130 | 87 (26.9%) | 1.089 (0.844, 1.404) | 0.513 |
| **Learning problem** | 89 (20.8%) | 0.784 (0.617, 0.996) | 0.046 | 97 (25.6%) | 0.975 (0.768, 1.237) | 0.833 |
| **Psychosomatic problem** | 74 (20.6%) | 0.797 (0.613, 1.038) | 0.092 | 65 (23.2%) | 0.905 (0.680, 1.204) | 0.492 |
| **Impulsive-hyperactive** | 115 (24.3%) | 1.026 (0.825, 1.277) | 0.815 | 69 (24.6%) | 0.973 (0.735, 1.287) | 0.847 |
| **Anxiety** | 70 (21.6%) | 0.842 (0.641, 1.104) | 0.213 | 65 (23.2%) | 0.884 (0.664, 1.176) | 0.396 |
| **Hyperactivity index** | 12 (16.7%) | 0.605 (0.324, 1.131) | 0.097 | 13 (25.5%) | 1.106 (0.580, 2.109) | 0.762 |

* The reference group was children who were not exclusively breastfed for first 6 months.

† Adjusted for birth weight (g), birth length (cm), preterm birth status, whether the child was an only child, parents’ marital status, education level, family income, parity, parental age at the time of the child's birth, supplementation time (month), and gestational diseases (gestational hypertension, preeclampsia/eclampsia and gestational diabetes mellitus).

**TableA 2 Risks of children’s PSQ scores based on feeding pattern (exclusive breastfeeding vs. non-** **exclusive breastfeeding) for first 6 months stratified by children’s sex^*†^.**

| **Items** | **Boys (9648)** | | **Girls (8219)** | |
| --- | --- | --- | --- | --- |
|  | **aβ (95%CI)** | ***P*** | **aβ (95%CI)** | ***P*** |
| **Conduct problem scores** | -0.009 (-0.023, 0.006) | 0.242 | 0 (-0.015, 0.014) | 0.961 |
| **Learning problem scores** | -0.001 (-0.019, 0.017) | 0.914 | -0.018 (-0.038, 0.001) | 0.070 |
| **Psychosomatic problem scores** | -0.007 (-0.015, 0.002) | 0.124 | -0.006 (-0.015, 0.003) | 0.192 |
| **Impulsive-hyperactive scores** | -0.012 (-0.031, 0.006) | 0.191 | -0.012 (-0.030, 0.007) | 0.209 |
| **Anxiety scores** | -0.007 (-0.020, 0.006) | 0.281 | -0.010 (-0.024, 0.004) | 0.164 |
| **Hyperactivity index scores** | -0.007 (-0.024, 0.009) | 0.370 | -0.010 (-0.026, 0.006) | 0.227 |

* The reference group was children who were not breastfed for first 6 months.

† Adjusted for birth weight (g), birth length (cm), preterm birth status, whether the child was an only child, parents’ marital status, education level, family income, parity, parental age at the time of the child's birth, supplementation time (month), and gestational diseases (gestational hypertension, preeclampsia/eclampsia and gestational diabetes mellitus).

**TableA 3 Risks of children’s behavioral problems based on overall breastfeeding time stratified by children’s sex^†^.**

| **Items** | **Boys (9648)** | | | | | | **Girls (8219)** | | | | | |
| --- | --- | --- | --- | --- | --- | --- | --- | --- | --- | --- | --- | --- |
|  | **7-12 months** | | | **≥13 months** | | | **7-12 months** | | | **≥13 months** | | |
|  | **Events** | **aOR (95%CI)** | ***P*** | **Events** | **aOR (95%CI)** | ***P*** | **Events** | **aOR (95%CI)** | ***P*** | **Events** | **aOR (95%CI)** | ***P*** |
| **Behavioral problems** | 578 (41.1%) | 0.907 (0.800, 1.028) | 0.126 | 224 (15.9%) | 0.751 (0.636, 0.887) | < 0.001 | 479 (41.7%) | 0.988 (0.859, 1.136) | 0.862 | 210 (18.3%) | 0.970 (0.811, 1.159) | 0.737 |
| **Conduct problem** | 104 (37.8%) | 0.792 (0.607, 1.033) | 0.085 | 43 (15.6%) | 0.736 (0.516, 1.050) | 0.090 | 138 (42.7%) | 1.093 (0.851, 1.405) | 0.486 | 61 (18.9%) | 1.118 (0.814, 1.538) | 0.491 |
| **Learning problem** | 183 (42.9%) | 0.981 (0.793, 1.213) | 0.857 | 66 (15.5%) | 0.730 (0.546, 0.976) | 0.034 | 159 (42%) | 1.083 (0.857, 1.369) | 0.504 | 79 (20.8%) | 1.180 (0.886, 1.571) | 0.258 |
| **Psychosomatic problem** | 139 (38.6%) | 0.840 (0.664, 1.062) | 0.145 | 59 (16.4%) | 0.786 (0.577, 1.070) | 0.126 | 119 (42.5%) | 0.981 (0.753, 1.277) | 0.885 | 44 (15.7%) | 0.812 (0.568, 1.160) | 0.252 |
| **Impulsive-hyperactive** | 194 (40.9%) | 0.905 (0.738, 1.108) | 0.333 | 71 (15%) | 0.757 (0.573, 1.001) | 0.051 | 116 (41.4%) | 0.909 (0.700, 1.181) | 0.476 | 41 (14.6%) | 0.770 (0.535, 1.107) | 0.158 |
| **Anxiety** | 132 (40.7%) | 0.937 (0.732, 1.199) | 0.604 | 56 (17.3%) | 0.885 (0.641, 1.222) | 0.459 | 115 (41.1%) | 0.918 (0.706, 1.194) | 0.522 | 42 (15%) | 0.752 (0.524, 1.079) | 0.122 |
| **Hyperactivity index** | 29 (40.3%) | 0.77 (0.468, 1.265) | 0.302 | 8 (11.1%) | 0.479 (0.220, 1.049) | 0.063 | 22 (43.1%) | 0.970 (0.537, 1.751) | 0.918 | 4 (7.8%) | 0.398 (0.136, 1.160) | 0.091 |

† Adjusted for birth weight (g), birth length (cm), preterm birth status, whether the child was an only child, parents’ marital status, education level, family income, parity, parental age at the time of the child's birth, supplementation time (month), and gestational diseases (gestational hypertension, preeclampsia/eclampsia and gestational diabetes mellitus).

**TableA 4 Risks of children’s PSQ scores based on overall breastfeeding time stratified by children’s sex^†^.**

| **Items** | **Boys (9648)** | | | | **Girls (8219)** | | | |
| --- | --- | --- | --- | --- | --- | --- | --- | --- |
|  | **7-12 months (n=3947)** | | **≥13 months (n = 1911)** |  | **7-12 months (n=3428)** |  | **≥13 months (n = 1566)** |  |
|  | **aβ (95%CI)** | ***P*** | **aβ (95%CI)** | ***P*** | **aβ (95%CI)** | ***P*** | **aβ (95%CI)** | ***P*** |
| **Conduct problem scores** | -0.011 (-0.025, 0.003) | 0.116 | -0.009 (-0.026, 0.009) | 0.325 | 0.01 (-0.005, 0.024) | 0.191 | 0.003 (-0.015, 0.021) | 0.745 |
| **Learning problem scores** | 0.006 (-0.011, 0.024) | 0.472 | -0.024 (-0.046, -0.003) | 0.028 | -0.008 (-0.027, 0.011) | 0.420 | -0.013 (-0.037, 0.012) | 0.310 |
| **Psychosomatic problem scores** | -0.008 (-0.016, 0) | 0.062 | -0.013 (-0.023, -0.003) | 0.012 | 0 (-0.008, 0.009) | 0.956 | -0.002 (-0.013, 0.009) | 0.756 |
| **Impulsive-hyperactive scores** | -0.014 (-0.032, 0.004) | 0.132 | -0.032 (-0.054, -0.010) | 0.005 | 0 (-0.018, 0.018) | 0.983 | -0.015 (-0.037, 0.008) | 0.210 |
| **Anxiety scores** | -0.001 (-0.017, 0.014) | 0.895 | -0.014 (-0.026, -0.001) | 0.034 | 0 (-0.013, 0.014) | 0.963 | -0.008 (-0.026, 0.009) | 0.332 |
| **Hyperactivity index scores** | -0.013 (-0.029, 0.002) | 0.098 | -0.017 (-0.036,0.003) | 0.090 | 0.007 (-0.008, 0.023) | 0.364 | -0.004 (-0.024, 0.016) | 0.697 |

† Adjusted for birth weight (g), birth length (cm), preterm birth status, whether the child was an only child, parents’ marital status, education level, family income, parity, parental age at the time of the child's birth, supplementation time (month), and gestational diseases (gestational hypertension, preeclampsia/eclampsia and gestational diabetes mellitus).

**Table A5 Characteristics of the participants of different genders**

| **Characteristics** | **Overall (n = 17867)** | **Boys (n = 9648)** | **Girls**  **(n = 8219)** | ***p*** |
| --- | --- | --- | --- | --- |
| Child age (mean (SD)) | 3.48 (0.27) | 3.48 (0.27) | 3.47 (0.26) | 0.012 |
| Birth weight (mean (SD)) | 3401.09 (864.35) | 3453.42 (884.55) | 3339.66 (835.90) | <0.001 |
| Birth length (mean (SD)) | 50.95 (5.55) | 51.10 (5.59) | 50.77 (5.50) | 0.001 |
| Maternal age at child birth (mean (SD)) | 29.23 (4.20) | 29.21 (4.22) | 29.24 (4.17) | 0.653 |
| Paternal age at child birth (mean (SD)) | 34.80 (4.68) | 34.80 (4.66) | 34.80 (4.70) | 0.977 |
| Maternal education level |  |  |  | <0.001 |
| Junior high school or lower | 4835 (27.1) | 2731 (28.3) | 2104 (25.6) |  |
| High school | 7340 (41.1) | 3903 (40.5) | 3437 (41.8) |  |
| College or higher | 5692 (31.9) | 3014 (31.2) | 2678 (32.6) |  |
| Paternal education level |  |  |  | 0.029 |
| Junior high school or lower | 4529 (25.3) | 2499 (25.9) | 2030 (24.7) |  |
| High school | 6237 (34.9) | 3398 (35.2) | 2839 (34.5) |  |
| College or higher | 7101 (39.7) | 3751 (38.9) | 3350 (40.8) |  |
| Family income (RMB/month) |  |  |  | 0.044 |
| <5000 | 4102 (23.0) | 4102 (23.0) | 2292 (23.8) |  |
| 5001-10000 | 6404 (35.8) | 6404 (35.8) | 3444 (35.7) |  |
| 10001-20000 | 3870 (21.7) | 3870 (21.7) | 2056 (21.3) |  |
| >20000 | 3491 (19.5) | 3491 (19.5) | 1856 (19.2) |  |
| Marital status |  |  |  | 0.915 |
| Married | 17353 (97.1) | 9375 (97.2) | 7978 (97.1) |  |
| Single | 514 (2.9) | 273 (2.8) | 241 (2.8) |  |
| Single child or not |  |  |  | 0.227 |
| No | 10014 (56.0) | 5367 (55.6) | 4647 (56.5) |  |
| Yes | 7853 (44.0) | 4281 (44.4) | 3572 (43.5) |  |
| Threatened abortion |  |  |  | 0.537 |
| No | 15420 (86.3) | 8312 (86.2) | 7108 (86.5) |  |
| Yes | 2447 (13.7) | 1336 (13.8) | 1111 (13.5) |  |
| Gestational hypertension |  |  |  | 0.775 |
| No | 17472 (97.8) | 9438 (97.8) | 8034 (97.7) |  |
| Yes | 395 (2.2) | 210 (2.2) | 185 (2.3) |  |
| Preeclampsia/ eclampsia |  |  |  | 0.906 |
| No | 17769 (99.5) | 9594 (99.4) | 8175 (99.5) |  |
| Yes | 98 (0.5) | 54 (0.6) | 44 (0.5) |  |
| Gestational diabetes mellitus |  |  |  | 0.252 |
| No | 16572 (92.8) | 8969 (93.0) | 7603 (92.5) |  |
| Yes | 1295 (7.2) | 679 (7.0) | 616 (7.5) |  |
| Preterm birth |  |  |  | 0.094 |
| No | 16304 (91.3) | 8772 (90.9) | 7532 (91.6) |  |
| Yes | 1563 (8.7) | 876 (9.1) | 687 (8.4) |  |
| Time of complementary food (mean (SD)) | 5.97 (2.36) | 5.97 (2.37) | 5.98 (2.34) | 0.896 |
| The duration of exclusive breastfeeding (mean (SD)) | 3.30 (3.14) | 3.25 (3.10) | 3.37 (3.20) | 0.01 |
| Exclusive breastfeeding in the first 6 months of life |  |  |  | 0.032 |
| No | 13347 (74.7) | 7270 (75.4) | 6077 (73.9) |  |
| Yes | 4520 (25.3) | 2378 (24.6) | 2142 (26.1) |  |
| The duration of breastfeeding (mean (SD)) | 8.70 (5.72) | 8.73 (5.75) | 8.67 (5.69) | 0.473 |
| Overall breastfeeding time |  |  |  | 0.371 |
| ≤6 moths | 7015 (39.3) | 3790 (39.3) | 3225 (39.2) |  |
| 7-12 moths | 7375 (41.3) | 3947 (40.9) | 3428 (41.7) |  |
| ≥13 moths | 3477 (19.5) | 1911 (19.8) | 1566 (19.1) |  |
